# Active DNA demethylation in human postmitotic cells correlates with activating histone modifications, but not transcription levels

**DOI:** 10.1186/gb-2010-11-6-r63

**Published:** 2010-06-18

**Authors:** Maja Klug, Sven Heinz, Claudia Gebhard, Lucia Schwarzfischer, Stefan W Krause, Reinhard Andreesen, Michael Rehli

**Affiliations:** 1 Department of Hematology, University Hospital Regensburg, Franz-Josef-Strauß-Allee 11, 93042 Regensburg, Germany; 2 Department of Cellular and Molecular Medicine, University of California, San Diego, 9500 Gilman Drive, La Jolla, CA 92093, USA; 3 Department of Internal Medicine 5, Hematology/Oncology, University of Erlangen-Nuernberg, Krankenhausstraße 12, 91054 Erlangen, Germany

## Abstract

**Background:**

In mammals, the dynamics of DNA methylation, in particular the regulated, active removal of cytosine methylation, has remained a mystery, partly due to the lack of appropriate model systems to study DNA demethylation. Previous work has largely focused on proliferating cell types that are mitotically arrested using pharmacological inhibitors to distinguish between active and passive mechanisms of DNA demethylation.

**Results:**

We explored this epigenetic phenomenon in a natural setting of post-mitotic cells: the differentiation of human peripheral blood monocytes into macrophages or dendritic cells, which proceeds without cell division. Using a global, comparative CpG methylation profiling approach, we identified many novel examples of active DNA demethylation and characterized accompanying transcriptional and epigenetic events at these sites during monocytic differentiation. We show that active DNA demethylation is not restricted to proximal promoters and that the time-course of demethylation varies for individual CpGs. Irrespective of their location, the removal of methylated cytosines always coincided with the appearance of activating histone marks.

**Conclusions:**

Demethylation events are highly reproducible in monocyte-derived dendritic cells from different individuals. Our data suggest that active DNA demethylation is a precisely targeted event that parallels or follows the modification of histones, but is not necessarily coupled to alterations in transcriptional activity.

## Background

The methylation of cytosine in the context of CpG dinucleotides in mammalian DNA is generally associated with gene silencing. The controlled setting and removal of DNA methylation are crucial for proper execution of essential regulatory programs in embryonic development, X-chromosome inactivation, parental imprinting as well as cellular differentiation [1-4]. Altered levels of cytosine methylation are associated with various diseases and may promote neoplastic development [5,6].

Whereas the process of DNA methylation, which is catalyzed by a group of DNA methyltransferases (DNMTs) is well characterized [7,8], the mechanisms responsible for the removal of methylated cytosines are less well understood. The failure of maintenance DNMTs to methylate a newly synthesized daughter strand during cell cycle progression represents a non-enzymatic, passive way of erasing the 5-methylcytosine mark that requires at least two cycles of replication for complete DNA demethylation. The documented existence of replication-independent DNA demethylation processes implies the presence of demethylating enzymes that actively remove either the methyl group, the methylated cytosine or whole nucleotides [9]. In flowering plants, the enzymes driving the active demethylation process are well known. *DME *(Demeter) and *ROS1 *(Repressor of silencing 1) are 5-methylcytosine glycosylases/lyases [10-12] that catalyze the first step of an active demethylation process that is linked to base excision repair. In animal cells, DNA demethylation through DNA repair mechanisms was first described by Jost and colleagues [13], who reported evidence for an enzymatic system replacing 5-methylcytosine by cytosine. Nuclear extracts from chicken embryos promoted demethylation of selective mCpGs in hemimethylated DNA through the formation of specific nicks 5' of 5-methyldeoxycytidine [13]. The responsible enzyme was later identified as a thymine DNA glycosylase [14]. Recently, it was shown that loss of methylation at an estrogen-responsive element coincides with the recruitment of DNMT3a/b, thymine DNA glycosylase and other base excision repair enzymes, confirming the implication of base excision repair [15]. The authors of the latter study assigned deaminating activities to both DNMTs; however, the involvement of DNMTs in catalyzing cytosine deamination remains controversial [9,16]. Another recent study showed that the hormone-regulated DNA demethylation of a gene promoter is mediated by glycosylase activity of MBD4 (methyl-CpG binding domain protein 4), another thymine glycosylase involved in removing T/G mismatches [17].

Most studies in the field of active DNA demethylation are based on cell models that normally proliferate, including pharmacologically arrested cell lines, primordial germ cells, and zebrafish or *Xenopus laevis *embryos, and this property is often utilized to argue in favor of passive mechanisms as a basis for the observed demethylation events.

In this study, the differentiation of human peripheral blood monocytes to immature dendritic cells (DCs) was used to analyze active demethylation processes. Peripheral blood monocytes are non-dividing progenitors of the mononuclear phagocyte system that are able to differentiate into morphologically and functionally divergent effector cells, including antigen presenting DCs, macrophages or osteoclasts [18]. Due to their proliferation-independent differentiation, human monocytes represent an excellent model to study active DNA demethylation. Global promoter experiments and fine-mapping studies revealed a considerable number of targeted, active demethylation events during monocyte to DC differentiation that were neither restricted to promoter regions nor generally associated with transcriptional changes. Irrespective of their genomic localization, DNA demethylation always coincided with the appearance of activating histone marks, suggesting a close association of chromatin modifying complexes with the DNA demethylation machinery.

## Results

### Differentiation of monocytes into myeloid dendritic cells occurs in the absence of proliferation

**Figure 1:**
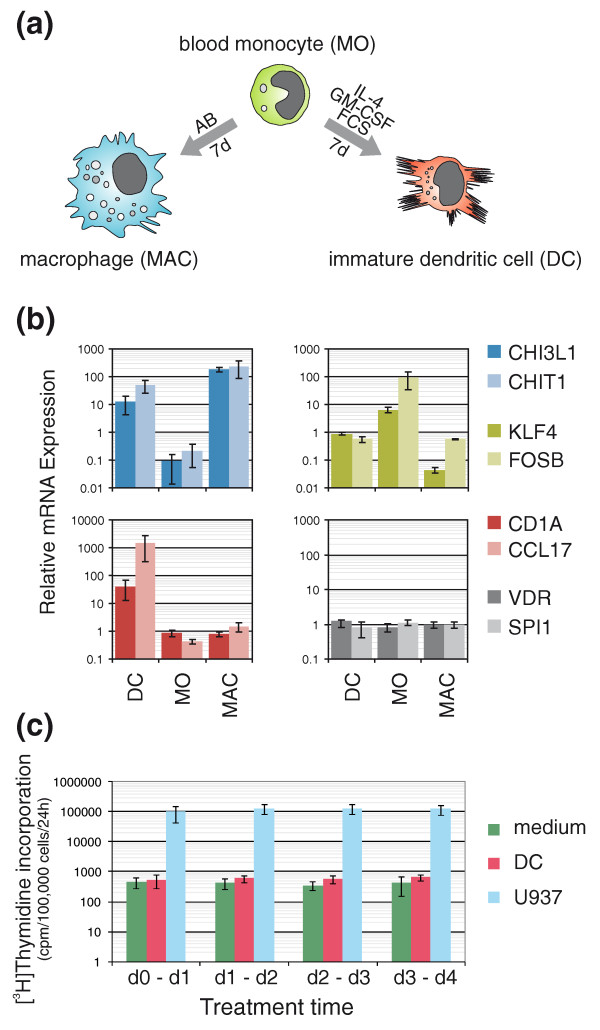
**Postproliferative differentiation model. (a) **Schematic presentation of the culture system. After leukapheresis and subsequent elutriation, monocytes (MO) were cultured either in the presence of IL-4, granulocyte-macrophage colony-stimulating factor (GM-CSF) and FCS to generate DCs or with human AB-serum to obtain macrophages (MAC) for 7 days. **(b) **Microarray expression profiles of several marker genes that are preferentially expressed in macrophages (*CHI3L1*, *CHIT1*), monocytes (*KLF4*, *FOSB*) or DCs (*CD1A*, *CCL17*) and control genes (*VDR*, *SPI1*) showing constant mRNA levels during differentiation. Shown are median-normalized microarray signal intensities derived from ten (monocytes) or six (DCs and macrophages) independent donors. **(c) **DCs and U937 cells were cultured with [^3^H]-thymidine for 20 h at different time points (day 0 to 1, day 1 to 2, day 2 to 3, day 3 to 4) during culture. Values represent mean ± standard deviation of three independent experiments. The U937 leukemia cell line served as positive control showing high thymidine incorporation levels.

Peripheral blood monocytes are characterized by a unique phenotypic plasticity and are able to differentiate into a number of morphologically and functionally diverse cell types *in vivo*, including the wide range of heterogeneous tissue macrophages, myeloid DCs and multinucleated osteoclasts. The distinct differentiation pathways can be recapitulated *in vitro*: culturing purified human monocytes for several days in the presence of human serum results in the generation of macrophages (Figure 1a), whereas they develop into myeloid DCs in the presence of the granulocyte-macrophage colony-stimulating factor and IL-4 [19]. Both cell types are characterized by a unique transcriptome (examples of marker gene expression are shown in Figure 1b) and their development from primary monocytes proceeds without cell division [20,21]. To confirm the absence of proliferation-dependent or -independent DNA synthesis, we measured the incorporation of [^3^H]-thymidine during the first 4 days of monocyte to DC differentiation. As shown in Figure 1c, we did not detect significant nucleotide incorporation during the analyzed time period. Similar results were obtained using 5-Bromo-2'-deoxy-uridine (BrdU) incorporation and subsequent immunostaining (Figure S1 in Additional file 1). In line with several earlier studies [22,23,25], proliferative activity ranged between 0 and 2% during the first 3 days of culture depending on the donor. Differentiating monocytes thus present an ideal post-mitotic cellular model to study epigenetic processes.

### Global identification of differentially methylated regions in dendritic cells and macrophages

**Figure 2:**
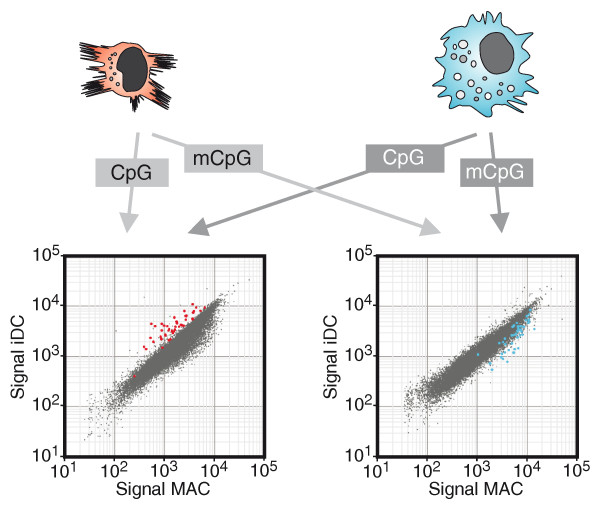
**Identification of differentially DNA methylated regions**. The fragmented genomes of macrophages (MAC) and immature dendritic cells (iDC) are separated into unmethylated (CpG) and methylated (mCpG) pools. Each pool is directly labeled using fluorescent dyes and each pool of one cell type is compared to the corresponding pool of the other cell type on a global promoter microarray. Microarray images are analyzed in combination to identify regions that show a reciprocal hybridization behavior. Representative scatter plots of CpG and mCpG pool hybridizations are shown. Probes enriched in the unmethylated pool of iDCs (red spots) were enriched in the methylated pool of macrophages (blue spots) and indicated the presence of DNA methylated regions. The reciprocal signal intensity ratios served as internal control for the reliability of microarray data.

**Figure 3:**
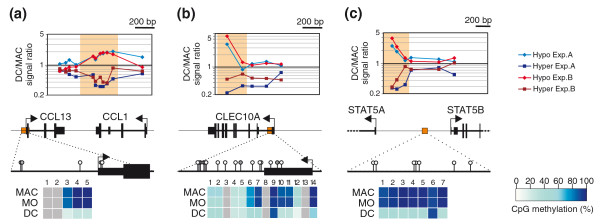
**Comparison of MCIp microarray and MassARRAY EpiTYPER data. (a-c) **Diagrams at the top show signal ratios of microarray probes for both independent experiments (donor A in blue, donor B in red) corresponding to their chromosomal localization. Typical DMRs are enriched in the hypomethylated fraction of one cell type and in the hypermethylated region of the other one, resulting in a mirror inverted image. Orange-colored zones indicate sequence regions validated via bisulfite conversion. Middle panels schematically present the chromosomal location of DMRs (orange boxes). Regions analyzed by MALDI TOF MS of bisulfite-converted DNA are indicated at the bottom. White circles represent detectable CpGs while grey circles (or grey boxes in the heat map below) show CpGs not measured by MS. Heat maps depict the methylation status of individual CpGs as shades of blue with each box representing a single CpG. Data of at least six independent donors were averaged.

**Figure 4:**
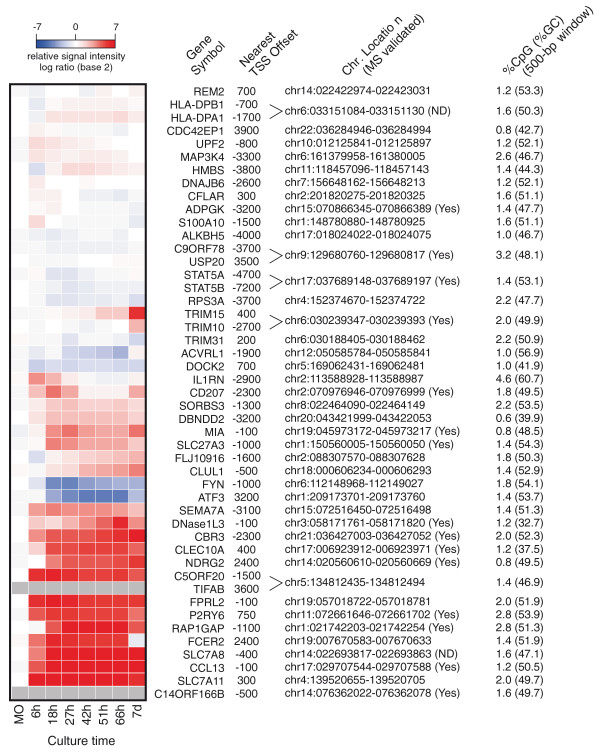
**mRNA expression profiles of genes associated with DMRs during DC differentiation**. Microarray expression levels of genes showing DC-specific CpG demethylation are displayed as a heat map. Blue, white and red represent low, medium and high expression, respectively. Data of two (DC day 7), three (DC 6 to 66 h) or six monocyte (MO) independent donors were averaged and normalized to monocyte samples. Distances from transcription start sites (TSSs) of neighboring genes, chromosomal locations (NCBI build 35/hg17) of the central DMR microarray probes and CpG as well as GC content in a 500-bp window are given on the right.

In order to assess occurrence and extent of active DNA demethylation during monocytic differentiation, we performed genome-wide methylation analyses using methyl-CpG immunoprecipitation (MCIp), a fractionation technique that is based on the salt concentration-dependent affinity of methylated and non-methylated DNA fragments towards an MBD-Fc fusion protein [26,27]. We refined and adapted the MCIp approach (schematically shown in Figure S2A in Additional file 1) for global promoter methylation analyses as recently described [28]. DNA samples from *in vitro*-differentiated monocyte-derived macrophages and DCs were separated into methylated (mCpG) and unmethylated (CpG) pools via MCIp (Figure S2B in Additional file 1; two biological replicates). Cell type-specific differences in the DNA methylation pattern were then identified by co-hybridization of either both hypermethylated or both hypomethylated DNA subpopulations to custom-designed 244 K human promoter oligonucleotide arrays (Figure 2) covering 5-kb regions around 17,000 known promoters of protein-coding genes. DNA fragments enriched in the methylated fraction of a given cell type are depleted in the corresponding unmethylated fraction. Therefore, the signal intensities in CpG pool and mCpG pool hybridizations complement each other ('mirror-image' approach; Figure 2; Figure S2 in Additional file 1) and allow the identification of differentially methylated regions (DMRs). In total, the microarray analyses revealed 45 regions that were hypomethylated in DCs compared to macrophages. In line with previous findings, most DMRs were of low CpG content and all residual sites were of intermediate CpG content (data not shown). To validate and quantify methylation differences, 28 representative regions (including 21 DMRs, 6 control regions selected from array results and one additional region) were selected for matrix-assisted laser desorption/ionisation-time of flight (MALDI-TOF) mass spectrometry (MS) analysis of bisulfite-treated DNA (for information on amplicons and MALDI-TOF MS results for all samples see Additional files 2 and 3). In total, 22 out of 25 regions detected with both assays (88%) were concordant between MCIp-microarray and MALDI TOF MS data. Figure 3 and Figure S3 in Additional file 1 show several examples for the high consistency of both approaches. Classical bisulfite-sequencing experiments of three representative regions also confirmed the targeted and reproducible demethylation of defined CpG residues in DCs (Figure S4 in Additional file 1). An annotated, complete list of DMRs is given in Figure 4, which also provides the position of each DMR relative to the transcription start site (TSS) of neighboring genes, local CpG/GC content, as well as corresponding mRNA expression data. Interestingly, DMRs were always methylated in monocytes, indicating that all observed methylation differences resulted from demethylation. We did not observe a single case of differentiation-associated *de novo *DNA methylation. Thus, most (if not all) DMRs are actively demethylated during DC differentiation.

### Active demethylation is targeted, not confined to proximal promoters and frequently but not imperatively linked with changes in transcription levels

The positional annotation of DMRs in Figure 4 demonstrates that active demethylation processes were not limited to proximal promoter regions. Regardless of genomic localization, demethylation of DMR proceeded in a highly reproducible fashion during monocyte differentiation using cells from several different individuals, as exemplified by the promoter-proximal *CCL13 *DMR, the promoter-distal, intergenic *CD207 *DMR and the intragenic *CLEC10A *DMR in Figure S5 in Additional file 1. The high reproducibility between different donors suggests that active CpG demethylation is a strictly targeted, non-random event. Furthermore, active DNA demethylation processes did not proceed synchronously during monocyte to DC development, with some CpGs being demethylated early (between 18 and 42 h) and others considerably later (> 51 h) (Figure S5 in Additional file 1 and data not shown). Most DMRs contained CpGs that were demethylated during the first 51 h, a period during which we never observed significant proliferation of DCs. The reproducibility of CpG demethylation and the presence of DMR-specific demethylation kinetics suggest sequence-specific targeting mechanisms that are likely mediated through DNA-binding factors either directly or indirectly.

We also correlated the presence of DMR with mRNA expression data obtained by whole genome microarray analyses of monocyte differentiation time courses (three biological replicates). As shown in Figure 4, about half of the DMR-associated genes were up-regulated during monocyte to DC differentiation. As a prime example for active demethylation at a proximal promoter, the *CCL13 *DMR was studied in more detail as shown in Additional file 1 (detailed characterization of the CCL13 promoter and Figure S6). The data suggest that CpG methylation in this particular case may contribute to transcription repression by preventing the binding of a yet unknown nuclear factor and that active demethylation at this site may be necessary for high level transcription in DCs. However, a correlation between demethylation and increased transcription was not universally observed as transcription levels of many DMR-associated genes remained largely unchanged during differentiation, as measured by microarray analysis. Increases in gene expression at demethylated genes also did not correlate with the local CpG or GC content, which was not significantly different between both groups of genes (*P *> 0.1, Mann Whitney U-test).

### Active DNA demethylation coincides with the appearance of active histone marks

**Figure 5:**
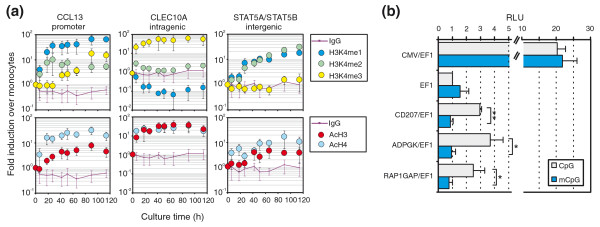
**Functional analyses of DMRs. (a) **Analysis of histone modifications across DMRs using ChIP. Chromatin was prepared at the indicated time points and precipitated against monomethyl histone H3 lysine 4 (H3K4me1), dimethyl histone H3 lysine 4 (H3K4me2) and trimethyl histone H3 lysine 4 (H3K4me3) as well as against acetylated histones H3 and H4 (AcH3 and AcH4). The IgG background level is indicated by the violet line. DNA enrichment of the indicated time points is normalized to 5% input DNA and shown relative to monocyte (0 h) enrichment. Data represent mean values ± standard deviation of at least three independent ChIP experiments. **(b) **Selected regions were cloned upstream of a basic EF1 promoter into the CpG-free luciferase vector pCpGL. The indicated plasmids were *in vitro *SssI-methylated (mCpG) or unmethylated (CpG) and transiently transfected into THP-1 cells. Luciferase activity was normalized against the activity of a co-transfected *Renilla *construct and mean values ± standard deviation (n = 3) are shown relative to the unmethylated pCpGL-EF1 construct. Asterisks indicate significant differences between methylated and unmethylated plasmids (*P < 0.05 and **P < 0.01, paired Student's *t*-test). RLU, relative light units.

Previous studies in other systems suggested a strong link between lineage-specific CpG demethylation events and changes in activating histone marks, including histone H3 lysine 4 (H3K4) methylation [28-30]. Since the above studies were done in proliferating cells, it was unclear whether the observed demethylation processes were active or passive. To determine whether similar correlations exist in a setting of post-proliferative monocytes that can only actively demethylate cytosine residues, we performed chromatin immunoprecipitation (ChIP) time course experiments studying the dynamics of histone modifications at selected DMRs, representative of the three possible genomic positions relative to the TSS (proximal promoter/intergenic/intragenic). As shown in Figure 5a and Figure S7 in Additional file 1, all seven actively demethylated regions tested exhibited increased H3K4 methylation or H3/4 acetylation during differentiation. As expected, H3K4 trimethylation was exclusively measured close to transcription start sites (*CCL13*, *CLEC10A*, *DNASE1L3*, *P2RY6*), whereas promoter-distal sites only acquired H3K4 mono- and dimethylation, which represents a signature indicative of putative enhancers [31].

We next asked whether promoter-distal DMRs display enhancer activity. Properties of generic enhancers include their ability to increase transcriptional activity in a heterologous context, which can be studied using traditional reporter gene assays. We recently developed a reporter vector that completely lacks CpG dinucleotides [32] and utilized this system to test for heterologous enhancer activity of seven selected DMRs (*STAT5A*, *CD207*, *CBR3*, *ADPGK, RAP1GAP, ALKBH5, RPS3A*) that are located in intergenic areas (between -4,700 to -1,100 bp from the nearest TSS). Transient transfections were performed in untreated, myeloid THP-1 cells using unmethylated (CpG) or *in vitro Sss*I-methylated (mCpG) reporter plasmids. *STAT5A*, *CBR3, ALKBH5 *and *RPS3A *fragments did not show enhancer activity in THP-1 cells (data not shown), which may relate to the fact that this cell line lacks DC-specific transcriptional regulators. As shown in Figure 5b, the remaining regions (*ADPGK, CD207, RAP1GAP*) significantly enhanced the activity of the basal (CpG-free) EF1 promoter and completely lost enhancer activity when methylated, suggesting that their enhancing activity is critically dependent on their CpG methylation status.

## Discussion

Despite the fact that numerous reports have described active DNA demethylation, its existence in humans is still controversial [16]. With few exceptions, previous studies were performed in artificial cell systems such as (pharmacologically arrested) cell lines [15,33] or embryonic cells [34,35], thus not entirely excluding a passive mechanism underlying the observed CpG demethylation. In contrast, human primary monocytes undergo differentiation into functionally different effector cells in the absence of DNA synthesis [20-25,36]. Consequently, in this post-mitotic differentiation model, any loss of CpG methylation observed must be the result of an active demethylation process.

We have adapted our previously developed comparative methylation profiling technology (MCIp) [26-28,37] to perform a systematic global screen for actively demethylated regions utilizing a promoter-based tiling microarray platform. This approach identified many novel loci that undergo active demethylation. Subsequent MS-based fine-mapping analysis of CpG methylation [38] performed in monocytes, macrophages and DCs during the time course of differentiation clearly confirmed the results of our global screen, demonstrating that active DNA demethylation is a strictly targeted process with locus-specific kinetics being almost identical between all individuals studied. As observed in proliferating cell systems [28-30], active demethylation events are predominantly found at promoter-distal sites, are linked with the appearance of activating histone marks such as H3K4 methylation and in some cases harbor methylation-sensitive enhancer activity. The striking concordance of demethylation-associated properties in mitotic and postmitotic cell systems suggests that the active demethylation machinery may contribute to DNA methylation dynamics in both settings.

Although the observed DNA demethylation events clearly point to active enzymatic processes, the underlying mechanisms are not completely understood. Recent work by other groups suggests an involvement of DNA repair mechanisms in active DNA demethylation. Other studies implicated DNMTs (as deaminases) [15] and base excision repair enzymes [15,17]. However, the proposed deaminating role of DNMTs remains controversial [9,16,17], and inhibitors of DNMTs did not affect the active DNA methylation process in our system (data not shown). The T/G mismatch repair enzyme MBD4 exhibits increased repair activity for methylated cytosines after hormone-induced phosphorylation and was shown to be required for the hormone-dependent demethylation of the *CYP27B1 *gene, suggesting that cytosine deamination may not necessarily be required for demethylation [17]. Another study argued for a model in which the TATA box binding protein-associated factor TAF12 recruits Gadd45a (growth arrest and DNA-damage induced-a) and the nucleotide excision repair machinery to promoters, resulting in active DNA demethylation [39]. A generalized role for TAF12 in our postmitotic system, however, seems unlikely because demethylation events in differentiating monocytes are not limited to promoters (where TAF12 binding is usually detected). Gadd45 proteins, initially identified as stress-inducible factors implicated in cell cycle arrest, DNA repair as well as apoptosis [40,41], have repeatedly been implicated in linking DNA repair mechanisms with DNA demethylation [36,42,43]. Work by Rai and colleagues [36], for example, suggest that GADD45 promotes the deamination of 5-methylcytosine through activation-induced cytidine deaminase (AICDA), which is followed by MBD4-dependent base excision. A critical role for AICDA in active DNA demethylation was recently also demonstrated in the setting of nuclear reprogramming and the generation of induced pluripotent stem cells [44]. However, especially the *in vivo *role of GADD45a in DNA demethylation was questioned by other studies [45,46]. In our model, GADD45 proteins are dynamically regulated during DC development (Figure S8 in Additional file 1) whereas *AICDA *mRNA expression was observed neither in monocytes nor during DC differentiation (data not shown). Global mRNA expression analyses across the differentiation time course additionally revealed DNA repair-associated genes that are significantly regulated during DC development (Figure S8 in Additional file 1). However, a functional implication of those candidates in CpG demethylation processes remains to be elucidated. So far, we have been unable to detect the recruitment of thymine DNA glycosylase or MBD4 to demethylated sites using ChIP assays (data not shown). This may suggest that repair processes related to DNA demethylation are different from those associated with DNA damage. However, this may also relate to the observed broad time frame (> 24 h) in which non-synchronized DNA demethylation processes occur in culture. The fact that only few monocytes actually undergo demethylation at a given time point may prevent the detection of transient interactions between demethylation machinery components and DNA.

Although we are currently unable to provide a clear molecular mechanism for the observed active DNA methylation processes observed during DC differentiation, our data reveal a number of novel and interesting insights into the nature of this process. A common property of all tested demethylated regions is the appearance of activating histone marks, such as mono- and dimethylation of H3K4 or acetylation of histones H3 and H4 (Figure 5a; Figure S7 in Additional file 1). The strict association of DNA demethylation and histone marks that are also found at enhancer elements [28] argue for the recruitment of DNA-binding factors that direct histone methyl- and/or acetyl-transferases to these sites. This is also supported by our limited enhancer reporter assays, where three out of seven tested regions displayed methylation-sensitive enhancer activity in a myeloid cell line. It is possible that the same factors responsible for the modification of histones also recruit the DNA demethylation machinery. Since the setting of activating histone marks in differentiating monocytes precedes or parallels active DNA demethylation, the deposited marks may themselves be recognized by histone code-reading proteins associated with the DNA demethylation machinery.

## Conclusions

We provide a first global screen for active DNA demethylation and demonstrate that active DNA demethylation during the differentiation of human monocytes is a strictly targeted, highly reproducible process that is neither limited to promoter regions nor necessarily associated with detectable changes at the level of transcription. It is, however, tightly linked with 'activating' histone modifications, suggesting that the DNA demethylation machinery may be recruited as part of other chromatin-modifying processes associated with gene activation or transcriptional priming.

## Materials and methods

### Ethics statement

Collection of blood cells from healthy donors was performed in compliance with the Helsinki Declaration. All donors signed an informed consent. Blood sampling, the leukapheresis procedure and subsequent purification of peripheral blood monocytes was approved by the local ethical committee (reference number 92-1782 and 09/066c).

### Cells

Peripheral blood monocytes were separated by leukapheresis of healthy donors followed by density gradient centrifugation over Ficoll/Hypaque and subsequent counter current centrifugal elutriation in a J6M-E centrifuge (Beckman Coulter GmbH, Krefeld, Germany) as previously described [47]. Monocytes were > 85% pure as determined by morphology and expression of CD14 antigen. Supernatants of monocyte cultures were routinely collected and analyzed for the presence of IL-6, which was usually low, indicating that monocytes were not activated before or during elutriation. To generate immature DCs, 1 × 10^6 ^monocytes/ml were cultured in RPMI 1640 medium (Thermo Scientific, Bonn, Germany) supplemented with 10% FCS (Biowhittaker, Verviers, Belgium), 20 U/ml IL-4 (Promokine, Heidelberg, Germany) and 280 U/ml granulocyte-macrophage colony-stimulating factor (Berlex, Seattle, WA, USA). For generating macrophages, 1 × 10^6 ^monocytes/ml were seeded in RPMI 1640 medium (HyClone) supplemented with 2% human pooled AB-group serum (Cambrex IEP GmbH, Wiesbaden, Germany) and cultured on teflon foils. THP-1 (human monocytic leukemia cell line) and U937 cells (human leukemic monocyte lymphoma cell line) were grown in RPMI 1640 plus 10% FCS (PAA, Pasching, Austria). RPMI 1640 was routinely enriched with 2 mM L-glutamine (Biochrome, Berlin, Germany), MEM non-essential amino acids (Invitrogen, Darmstadt, Germany), sodium pyruvate (Invitrogen), MEM vitamins (Invitrogen), 50 U/ml penicillin/streptomycin (Invitrogen), and 50 nM 2-mercaptoethanol (Invitrogen). The human cervical carcinoma cell line HeLa was maintained in Dulbecco's modified Eagle's medium plus 10% FCS.

### DNA isolation

Genomic DNA was prepared using the Blood and Cell Culture Midi Kit from Qiagen (Hilden, Germany). DNA concentration was determined with the ND-1000 NanoDrop spectrophotometer (Thermo Scientific, Bonn, Germany) and quality was assessed by agarose gel electrophoresis.

### RNA isolation

Total cellular RNA was isolated using the RNeasy Mini Kit (Qiagen). RNA concentration was measured with the ND-1000 NanoDrop Spectrophotometer (Thermo Scientific) and quality was controlled on agarose gels or using the Agilent Bioanalyzer (Böblingen, Germany).

### Whole genome expression analysis

Labeling, hybridization and scanning of high quality RNA was performed using the Agilent microarray platform according to the manufacturer's instructions. In brief, 200 to 1,000 ng of high-quality RNA were amplified and Cyanine 3-CTP-labelled with the One colour Low RNA Input Linear Amplification Kit. Labeling efficiency was controlled using the NanoDrop spectrophotometer and 1.65 μg labeled cRNA were fragmented and hybridized on Whole Human Genome Expressionarrays (4 × 44 K Agilent). Microarrays were washed and subsequently scanned with an Agilent scanner. Raw data were extracted with Feature Extraction 9.5.1 software and analyzed using GeneSpring GX 10.0.2 (Agilent). Data were normalized to the 75th percentile and baseline-transformed to the median of freshly isolated monocyte samples. Microarray data have been submitted and are available from the National Center for Biotechnology Information (NCBI) Gene Expression Omnibus (GEO) repository (accession number [GEO:GSE19236]).

### Chromatin immunoprecipitation

Preparation of cross-linked chromatin was performed as described previously [48] with some modifications. Briefly, cells were treated with 1% formaldehyde solution for 7 minutes at room temperature and quenched by 0.125 M glycine. After washing with phosphate-buffered saline including 1 mM phenylmethylsulfonylfluoride, 2 × 10^6 ^cells were resuspended in 50 μl lysis buffer 1A (L1A: 10 mM HEPES/KOH pH 7.9, 85 mM KCl, 1 mM EDTA pH 8.0) and lysed by adding 50 μl lysis buffer 1B (L1A + 1% Nonidet P-40) for 10 minutes on ice. Cross-linked chromatin was sheared to an average DNA fragment size around 400 to 600 bp using a Branson Sonifier 250 (Danbury, CT, USA). After centrifugation, 4 μl of the lysate were used as 5% input. After pre-clearing with 50 μl Sepharose CL-4B beads (blocked with 0.2% bovine serum albumin and 5 μg sheared salmon sperm for 1 h at 4°C) for 2 h, chromatin samples were immunoprecipitated overnight with 2.5 μg rabbit anti-RNA polymerase II CTD repeat YSPTSPS (phospho S5), anti-monomethyl Histone H3 (Lys4) (ab5131, ab8895, respectively; Abcam, Cambridge, UK), anti-dimethyl-Histone H3 (Lys4), anti-trimethyl-histone H3 (Lys4), anti-acetyl-Histone H3, anti-acetyl-Histone H4 or anti-IgG (07-030, 05-745, 06-599, 06-866 or 12-370, respectively; Millipore, Schwalbach/Ts., Germany). Before precipitation, protein A-Sepharose beads (GE Healthcare, Munich, Germany) were treated with 2 μg sheared salmon sperm DNA for 1 h at 4°C. Immunocomplexes were then recovered by incubation for 2 h with the blocked beads at 4°C. After reverse cross-linking, DNA was purified using the QIAquick PCR purification kit (Qiagen) according to the manufacturer's instructions except that the samples were incubated with phosphate buffer for 30 minutes and that they were eluted with 100 μl elution buffer. Enrichment of specific DNA fragments in the immunoprecipitated material was determined by quantitative PCR on the Realplex Mastercycler using the Quantifast SYBR Green PCR Kit (Qiagen). Oligonucleotide sequences are given in Additional file 4.

### Methyl-CpG immunoprecipitation

Production of the recombinant MBD-Fc protein and MCIp were carried out as previously described [26,27] with modifications. Briefly, genomic DNA of DCs and macrophages was sonicated to a mean fragment size of 350 to 400 bp using a Branson Sonifier 250. Four micrograms of each sample were rotated with 200 μl protein A-Sepharose 4 Fast Flow beads (GE Healthcare) coated with 70 μg purified MBD-Fc protein in 2 ml Ultrafree-MC centrifugal devices (Millipore) for 3 h at 4°C in a buffer containing 250 mM NaCl (buffer A). Beads were centrifuged to recover unbound DNA fragments (250 mM fraction) and subsequently washed with buffers containing increasing NaCl concentrations (300, 350, 400, 450, 500 mM; buffers B to F). Densely CpG-methylated DNA was eluted with 1,000 mM NaCl (buffer G) and all fractions were desalted using the QIAquick PCR Purification Kit (Qiagen). The separation of CpG methylation densities of individual MCIp fractions was controlled by quantitative PCR using primers covering the imprinted *SNRPN *and a region lacking CpGs (*Empty*), respectively. Fractions containing unmethylated DNA (250 to 350 mM NaCl) or methylated DNA (400 to 1,000 mM NaCl) fractions were pooled before subsequent labeling.

### Promoter microarray handling and analysis

Unmethylated (CpG) and methylated (mCpG) pools of both cell types were labeled with Alexa Fluor 5-dCTP (DCs) and Alexa Flour 3-dCTP (macrophages) using the BioPrime Total Genomic Labeling System (Invitrogen) as indicated by the manufacturer. Hybridization on 244K Custom-Oligonucleotide-Microarrays (containing about 17,000 promoter regions (-4,000 to + 1,000 bp relative to the TSS) as well as few regions tiled over large genomic intervals)) and washing was performed as recommended by the manufacturer (Agilent). Images were scanned immediately using a DNA microarray scanner (Agilent) and processed using Feature Extraction Software 9.5.1 (Agilent) with a standard comparative genomic hybridization protocol (including linear normalization). Processed signal intensities were then imported into Excel 2007 for further analysis. Probes with abnormal hybridization behavior (extremely high or extremely low signal intensities in one of the channels) were excluded. To detect DMRs, log_10 _ratios of individual probes from both comparative genome pool hybridizations were subtracted. A more detailed description of the global methylation assay (MCIp and hybridization) is given in [28]. Microarray data have been submitted and are available from the NCBI GEO repository (accession number [GEO:GSE19395]).

### Mass spectrometry analysis of bisulfite-converted DNA

We chose a set of genomic regions based on the MCIp microarray results and designed 48 amplicons for bisulfite conversion. Genomic sequences were extracted from the UCSC genome browser [49] and PCR primers were designed using the Epidesigner web tool [50]. For each reverse primer, an additional T7 promoter tag for *in vitro *transcription was added, as well as a 10-mer tag on the forward primer to adjust for melting temperature differences. All primers were purchased from Sigma-Aldrich (Munich, Germany; for sequences see Additional file 2). Sodium bisulfite conversion was performed using the EZ DNA Methylation Kit (Zymo Research, Orange, CA, USA) with 1 μg of genomic DNA and an alternative conversion protocol (Sequenom, San Diego, CA, USA). Amplification of target regions was followed by treatment with shrimp alkaline phosphatase, reverse transcription and subsequent RNA base-specific cleavage (MassCLEAVE, Sequenom) as previously described [38]. Cleavage products were loaded onto silicon chips (spectroCHIP, Sequenom) and analyzed by MALDI-TOF MS (MassARRAY Compact MALDI-TOF, Sequenom). Methylation was quantified from mass spectra using the Epityper software v1.0 (Sequenom). Methylation ratios for all samples are given in Additional file 3

### Proliferation assay

Proliferation capacity of cells was measured using [^3^H]-thymidine incorporation. Cells were seeded in 96-well microtiter plates and pulsed with 0.5 μCi [methyl-^3^H]-thymidine per well (Hartmann Analytics, Braunschweig, Germany) for 20 h. Cells were harvested onto UniFilter plates using a Wallac harvester and incorporated [^3^H]-thymidine was determined with a Wallac Betaplate counter (all from PerkinElmer, Gaithersburg, MD, USA).

### Plasmid construction and transient DNA transfections

Differentially methylated regions (ranging from 800 to 1,000 bp) were PCR-amplified from human genomic DNA and cloned into the CpG-free pCpGL-CMV/EF1 vector [32] by ligation replacing the cytomegalovirus (CMV) enhancer with the DMRs. Primer sequences are given in Additional file 4. Inserts were verified by sequencing. Luciferase reporter constructs were either mock-treated or methylated *in vitro *with *Sss*I methylase for 4 h at 37°C and purified with the Plasmid Quick Pure Kit (Macherey-Nagel, Dueren, Germany) or using the Endofree Plasmid Kit (Qiagen). THP-1 and HeLa cells were transfected as described [51]. The transfected cells were cultivated for 48 h and harvested. Cell lysates were assayed for firefly and *Renilla *luciferase activity using the Dual Luciferase Reporter Assay System (Promega, Mannheim, Germany) on a Lumat LB9501 (Berthold Detection Systems GmbH, Pforzheim, Germany). Firefly luciferase activity of individual transfections was normalized against *Renilla *luciferase activity.

## Abbreviations

AICDA: activation-induced cytidine deaminase; bp: base pair; ChIP: chromatin immunoprecipitation; DC: dendritic cell; DMR: differentially methylated region; DNMT: DNA methyltransferase; FCS: fetal calf serum; GEO: Gene Expression Omnibus; H3K4: histone 3 lysine 4; IL: interleukin; MALDI-TOF: matrix-assisted laser desorption/ionisation-time of flight; MCIp: methyl-CpG-immunoprecipitation; MS: mass spectrometry; NCBI: National Center for Biotechnology Information; TSS: transcription start site.

## Authors' contributions

MK participated in study design, performed experiments, analyzed data and aided in the manuscript preparation, SH participated in study design, and performed and analyzed experiments, CG and LS performed experiments, SK and RA participated in study design, MR conceived and coordinated the study, analyzed data and drafted the manuscript. All authors read and approved the final manuscript.

## Supplementary Material

Additional file 1 **Supplementary methods, additional results, description of supplementary tables, and supplementary figures**. Click here for file

Additional file 2 **Oligonucleotides for bisulfite amplicon generation. Genomic locations and oligonucleotides for EpiTYPER bisulfite amplicons**. Click here for file

Additional file 3 **MassARRAY EpiTYPER results**. EpiTYPER methylation ratios of individual CpG units in 46 amplicons covering 26 distinct genomic locations are given for all samples of different donors along with mean values for d7 macrophages (MAC), monocytes (MO), dendritic cells (DC) at day 7 or 51 h and data for unmethylated, 33%, 66% and 100% methylated control DNA. Amplicons were grouped according to their microarray results: MCIp different (regions were detected as differentially methylated between MAC and DC; 18 regions in total, three are marked as false positive in the microarray experiments), MCIp marginally different (one region), MCIp ND (one region - CCL17 promoter - that was not present on the array but represented a possible target; MCIp control (regions that were detected as equally methylated (or unmethylated) between MACs and DCs, 6 regions). For two additional regions (HLA-DPB/A1 and SLC7A8), none of the tested amplicons worked. Click here for file

Additional file 4 **Oligonucleotide sequences used for quantitative PCR, cloning, and electrophoretic mobility shift assay**. Click here for file
