# Supplementary material for: Active DNA demethylation in human postmitotic cells correlates with activating histone modifications, but not transcription levels
Source: Genome Biol. 2010 Jun 18;11(6):R63. doi: 10.1186/gb-2010-11-6-r63 (PMC2911111; doi:10.1186/gb-2010-11-6-r63)
Supplement: Additional file 1 — Supplementary methods, additional results, description of supplementary tables, and supplementary figures. [file gb-2010-11-6-r63-S1.PDF]

Supplementary Material

for

Active DNA demethylation in human postmitotic cells correlates with activating histone modifications, but not transcription levels

Maja Klug[1], Sven Heinz[2], Claudia Gebhard[1], Lucia Schwarzfischer[1], Stefan W. Krause[3], Reinhard Andreesen[1], Michael Rehli[1#]

[1]Department of Hematology, University Hospital Regensburg,
93042 Regensburg,
Germany

[2]Department of Cellular and Molecular Medicine, University of California,
La Jolla, CA 92093,
USA

[3]Department of Internal Medicine 5, Hematology/Oncology,
University of Erlangen-Nuernberg,
91054 Erlangen,
Germany

Supplement Index:

Supplementary Methods page 02
Detailed characterization of the CCL13 promoter page 04
Description of Supplementary Tables S1-S3 page 05
Legends for Supplementary Figures S1-S8 page 06-08
Supplementary Figures S1-S8 page 09-16

Supplementary Methods

BrdU labelling

BrdU labelling and visualisation were performed using the 5-Bromo-2'-deoxy-uridine Labeling and Detection Kit II from Roche. In brief, dendritic cells and THP1 cells were labelled with 10 µM BrdU for 19 h or 66 h.. When the cells were incubated for 66 h, BrdU (10 µM) was supplemented to the culture every 24 hours. After washing, cells were resuspended in PBS (2 and 20×10^{6} cells/ml) and dropped onto adhesion chamber slides(MARIENFELD, Germany). Fixation and detection were carried out according to the manufacturer's instructions. Slides were then evaluated in a light microscope by counting BrdU-positive and –negative cells (100 cells per visual field, 4 fields of each sample in total).

Bisulfite sequencing

Bisulfite conversion of genomic DNA and subsequent PCR were performed as described for the mass spectrometry analysis. PCR products were then cloned into a pCR 2.1 TOPO vector using the TOPO TA Cloning kit from Invitrogen. Insert-containing plasmids of single colonies were sequenced (GENEART) and analyzed using GeneRunner software (http://www.generunner.net/).

Nuclear extracts and electrophoretic mobility shift assay

Nuclear extracts were prepared as described [1]. All buffers used contained 1 mM Na_3VO*4* and a cocktail of protease inhibitors (2 µg/ml aprotinin, 5 µg/ml leupeptin, 1 µg/ml pepstatin, 0.5 mg/ml pefabloc SC, 20 µg/ml E46, 50 µg/ml antipain, 160 µg/ml chymostatin). Double-stranded oligonucleotides were labeled with α-[32P] dGTP using Klenow fragment (Roche). The binding reaction contained 10 µg of nuclear extract protein, 0.5 µg of poly d(I/C), 20 mM HEPES pH 7.9, 60 mM KCl, 1mM DTT, 1mM EDTA, pH 8.0, 5% glycerol and 20 nmol of DNA probe in a final volume of 10 µl. After 15 min incubation at RT, samples were applied to the polyacrylamide gel buffered with 150 mM Tris pH 8.8. Gels were run at 160 V for 150 min with 1x TGE as electrode buffer, fixed in 5% acetic acid, dried and autoradiographed.

Quantitative RT-PCR

500 ng to 1 µg of total RNA were reverse transcribed using Superscript II MMLV-RT (Promega, Mannheim, Germany). Real Time PCR was performed on a Realplex Mastercycler EP (Eppendorf, Hamburg, Germany) using the Quantifast SYBR Green PCR Kit (Qiagen) as indicated by the manufacturer. Oligonucleotide sequences are listed in Table S3 in Additional file 4. To control specificity of the primers, melting curves were analysed. Expression data were normalized to expression of the *HPRT* housekeeping gene. The relative units were calculated from standard curve plotting serial log dilutions against the PCR cycle number (CP) at which the measured fluorescence intensity reached a fixed value. For each sample, data of at least three different donors were measured in duplicates and averaged.

Reporter plasmid construction

A 1.1 kb genomic fragment of the *CCL13* promoter was amplified from genomic DNA using the Expand High Fidelity PCR system (Roche Biochemicals) and primers given in Table S3 in Additional file 4. This fragment was cloned into the pGL3 reporter vector (Promega) and

deletions were generated by PCR using sense primers given in Supplementary Table S1 and the vector primer GL2 (Promega). Site-specific mutations were introduced using the same primers used for EMSA (see Table S3 in Additional file 4) and all inserts were verified by sequencing.

Additional antibodies used for chromatin immunoprecipitation (ChIP)

rabbit anti-STAT6 (sc-1698X, Santa Cruz Biotechnology, Inc.); anti-RNA polymerase II CTD repeat YSPTSPS (phospho S5). Note that lysis buffers were supplemented with phosphatase inhibitors (50 mM ß-glycerophosphate and 1 mM Na_2VO_4) when phosphorylated proteins had to be precipitated.

Detailed characterization of the CCL13 promoter

The proximal promoter of the DC-specific chemokine gene *CCL13* (chemokine (C-C motif) ligand 13; also known as MCP-4) was studied in more detail to obtain evidence for a functional role of DNA methylation during DC differentiation. *CCL13* was highly induced during the course of monocyte to dendritic cell differentiation (Figure S6A in Additional file 1). In parallel, DC-specific demethylation of promoter CpGs adjacent to the transcription start site (one single and two CpGs in tandem, located at -80 bp and -20 bp, respectively) was detected between 18 and 42 hours (Figure 3A and Figure S5 in Additional file 1). The proximal promoter contains several putative binding sites for transcription factors, including STAT motifs. Chromatin immunoprecipitation experiments revealed that the IL-4-induced transcription factor STAT6 as well as RNA polymerase II (Pol II) were recruited to the *CCL13* promoter with delayed kinetics relative to DNA demethylation (Figure S6B in Additional file 1), suggesting that additional events (e.g. chromatin remodelling and/or epigenetic reprogramming) may be required for *CCL13* transcription.

In order to characterise the effects of dendritic cell-specific CpG demethylation on nuclear protein binding to the *CCL13* promoter, we performed electrophoretic mobility shift assays with nuclear extracts from monocyte-derived macrophages and monocyte-derived dendritic cells using probes that encompass either the single (-80 bp) CpG motif upstream of the TATA box or the TATA box and the two CpG (+20 bp) motifs downstream (sequences are given in Table S3 in Additional file 4). While the latter oligonucleotide bound nuclear proteins of dendritic cells and macrophages either in the methylated or unmethylated state (data not shown), the single (-80 bp) CpG motif-containing oligonucleotide only complexed nuclear protein when unmethylated, again from both cellular sources (Figure S6C in Additional file 1 and data not shown). Competitor analysis revealed that the unmethylated probe was displaced exclusively by unmethylated oligonucleotide but not by its methylated counterpart, indicating methylation-sensitive binding of an unknown factor to the (-80 bp) CpG motif. A query using MatInspector (32) identified a putative core consensus binding site for the whn transcription factor at the CpG motif at -80 bp. Reversing the CpG (ACGC core to AGCC) or entirely eliminating the motif (ACGC to AGAG) almost completely abolished protein binding to the oligonucleotides (Figure S6D in Additional file 1). We next performed luciferase reporter assays in pro-monocytic THP-1 and HeLa cells using the 5'-proximal *CCL13* promoter showing that sequences beyond -73 bp contain myeloid-specific activity. As shown in the bottom panel of Figure S6E in Additional file 1, the wild-type -125 bp minimal promoter construct (-125 wt) showed significant reporter activity in these cells, and both plasmids carrying mutations in the –80 bp CpG motif exhibited only 50-60% promoter activity compared to the wild type promoter. These data suggest that CpG methylation at the *CCL13* promoter may contribute to transcription repression by preventing the binding of a yet unknown nuclear factor. In turn, active demethylation at this site may be necessary for high level transcription in DC.

Description of Supplementary Tables

Supplementary Table S1

Table S1 lists genomic locations and oligonucleotides for EpiTYPER bisulfite amplicons

Supplementary Table S2

Table S2 lists MassARRAY EpiTYPER results. EpiTYPER methylation ratios of individual CpG units in 46 amplicons covering 26 distinct genomic locations are given for all samples of different donors along with mean values for d7 macrophages (MAK), monocytes (MO), dendritic cells (DC) at day7 or 51h and data for unmethylated, 33%, 66% and 100% methylated control DNA. Amplicons were grouped according to their microarray results: MCIp different (regions were detected as differentially methylated between MAK and DC; 18 regions in total, three are marked as false positive in the microarray experiments), MCIp marginally different (one region), MCIp ND (one region - CCL17 promoter - that was not present on the array but represented a possible target; MCIp control (regions that were detected as equally methylated (or unmethylated) between MAC and DC, 6 regions). For two additional regions (HLA-DPB/A1 & SLC7A8), none of the tested amplicons worked.

Supplementary Table S3

Table S3 lists oligonucleotide sequences used for qPCR, cloning, and EMSA.

Supplementary Figure Legends

Figure S1

BrdU incorporation of monocyte-derived dendritic cells. Dendritic cells (DC) and THP-1 cells were cultured with 10 µM BrdU for 19 h at different time points (day 0–1, day 1-2, day 2-3, day 3-4) or for 66 h (day 0-3). (A) Photographic images of one donor and one THP1 kinetic are exemplarily shown (twentyfold magnification). (B) BrdU-positive cells were evaluated by counting 100 cells in each of four different visual fields. Values are mean ±SD of four independent experiments. The cell line THP-1 served as positive control demonstrating high BrdU incorporation levels.

Figure S2

Identification of differentially DNA methylated regions (DMR). (A) Schematic outline of the MCIp fragmentation and hybridisation strategy. The fragmented genomes of DC and MAC are separated into unmethylated (CpG) and methylated (mCpG) pools. Each pool is directly labeled using fluorescent dyes and each pool of one cell type is compared to the corresponding pool of the other cell type on a locus-wide microarray. Microarray images are compared to identify regions that show a reciprocal hybridization behavior. (B) Enrichment of methylated and unmethylated DNA was controlled by qPCR. The 250 mM, 300 mM and the 350 mM NaCl fraction and seperately all remaining fractions were combined to divide the genome of both donors into an unmethylated (CpG) and a methylated DNA (mCpG) pool, respectively. *SNRPN* is an example for an imprinted locus and the *Empty* region does not contain any CpG residue.

Figure S3

Comparison of MCIp microarray and MassARRAY EpiTYPER data. (A-D) Additional comparisons of both methods (microarray hybridisation and mass spectrometry of bisulfite converted DNA) as described in Figure 3. (D) Example for a region that is not affected by DNA methylation changes.

Figure S4

DNA demethylation measured using classical bisulfite sequencing. Bisulfite sequencing of representative promoter (A), inter- (B) and intragenic (C) regions. Panels on top schematically present the chromosomal location of DMRs (orange boxes). The position of CpGs within the amplicons is indicated by “lollipops”. The methylation status of individual CpGs is displayed by either white (unmethylated) or black (methylated) squares. Squares in grey indicate sequence errors. Lines of squares represent independently sequenced clones derived from two independent donors at two different time points each (0 h and 51 h).

Figure S5.

Reproducibility of DNA demethylation events Mass spectrometry analysis of bisulfite-converted DNA at the indicated differentiation time points. Heatmaps indicate the methylation content in blue gradations with each box representing a single CpG dinucleotide. Grey boxes indicate CpGs that were not detected by MALDI-TOF MS. Data of four individual donors are shown as heatmaps demonstrating the high reproducibility of methylation changes between independent experiments.

Figure S6

Characterisation of the *CCL13* promoter. ***(A)*** Expression profile of *CCL13* during the differentiation of monocytes (MO) into immature dendritic cells (DC) until day 7 (7d) compared to macrophages (MAC). Results were normalised for *HPRT* expression. Values are mean ± SD obtained from three independent experiments. ***(B)*** Chromatin immunoprecipitation (ChIP) for Pol II and the transcription factor STAT6. Grey spots indicate the IgG background level. Signals, specific for the *CCL13*-promoter region were normalised to the signals of an unbound upstream control region. Values are the mean ± SD of four independent experiments. ***(C)*** EMSA were performed using labeled unmethylated (CpG1*) or methylated (mCpG1*) oligonucleotide and nuclear extracts of monocyte-derived DC. For competition analysis, unlabeled oligonucleotides were added at 100-fold excess where indicated (+) above the respective lanes. Specific bands are marked with arrows and non-specific bands are marked with asterisks. ***(D)*** Binding of nuclear extract protein from monocyte-derived DC to labeled, unmethylated CpG1* oligonucleotide (abbreviated as C*) was only displaced by a 100-fold excess of unlabeled unmethylated CpG1 (C) but not methylated (mC), or mutated (M1, M2) CpG1 oligonucleotide. Specific bands are marked with arrows and non-specific bands are marked with asterisks. ***(E)*** Reporter assays using wild type or mutated reporter constructs of the *CCL13* promoter in myeloid THP-1 and HeLa cells. Luciferase activity is relative to the promoter-less pGL3 basic vector and values are the means + S.D. obtained from at least three independent experiments.

Figure S7.

Analysis of histone modifications across DMRs using ChIP. Chromatin was prepared at the indicated time points and precipitated against monomethyl H3 lysine 4 (H3K4me1), dimethyl H3 lysine 4 (H3K4me2) and trimethyl H3 lysine 4 (H3K4me3) as well as against acetylated histones H3 and H4 (AcH3 and AcH4). The IgG background level is indicated by the violet line. DNA enrichment of four additional DMR at the indicated time points is normalised to 5% input DNA and shown relative to monocyte (0 h) enrichment. Data represent mean values ±SD of at least three independent ChIP experiments.

Figure S8

Expression profiles of genes associated with DNA repair (A) Expression levels of genes showing at least 5 fold up- or down regulation are indicated by colouring. Blue, white and red represent low, medium and high expression, respectively. After “per chip” and ”per gene normalization”, data of two (168 h time point), three (6 h to 66 h time points) or six (monocytes, indicated as MO) independent donors were averaged and evaluated relative to

monocytes. Genes whose products were already associated with active DNA demethylation in literature are indicated in red. (B) Validation of mRNA microarray experiments using RT-qPCR. Real-time PCR for *GADD45* expression at the indicated differentiation time points of immature dendritic cells (iDC) compared to monocytes (MO) and macrophages (MAC) after 168h in culture. Results were normalised for *HPRT* expression. Values are means ± SD obtained from three independent experiments.

Supplementary References

1. Rehli M, Poltorak A, Schwarzfischer L, Krause SW, Andreesen R, Beutler B (2000) PU.1 and interferon consensus sequence-binding protein regulate the myeloid expression of the human Toll-like receptor 4 gene. *J Biol Chem* 275: 9773-9781.

Figure S1

A

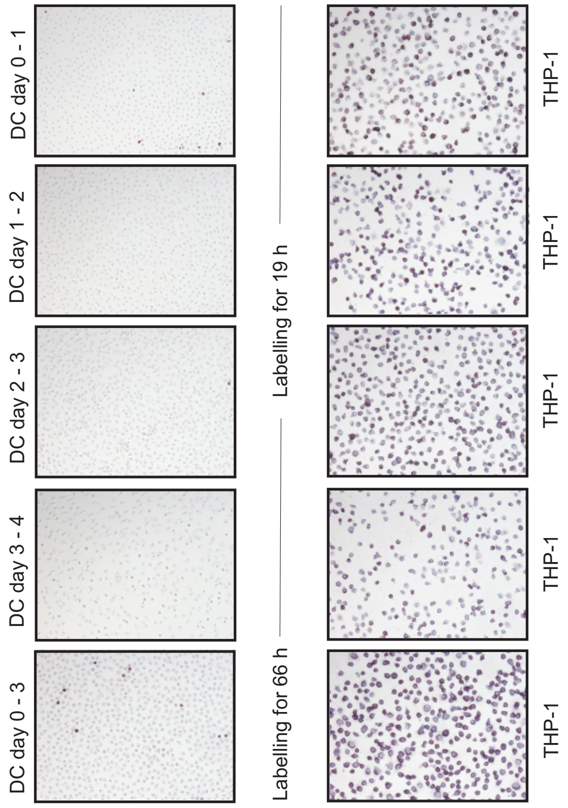

DC day 0 - 1
DC day 1 - 2
DC day 2 - 3
DC day 3 - 4
DC day 0 - 3
Labelling for 19 h
Labelling for 66 h
THP-1
THP-1
THP-1
THP-1
THP-1

B

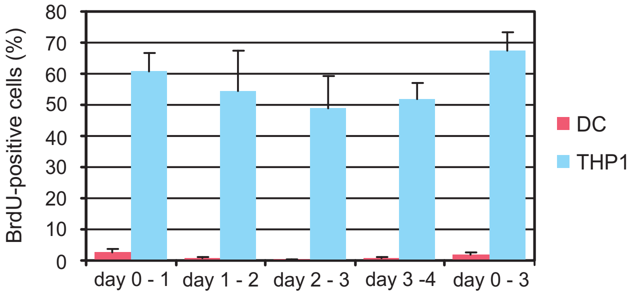

BrdU-positive cells (%)
80
70
60
50
40
30
20
10
0
day 0 - 1
day 1 - 2
day 2 - 3
day 3 -4
day 0 - 3
DC
THP1

Figure S2

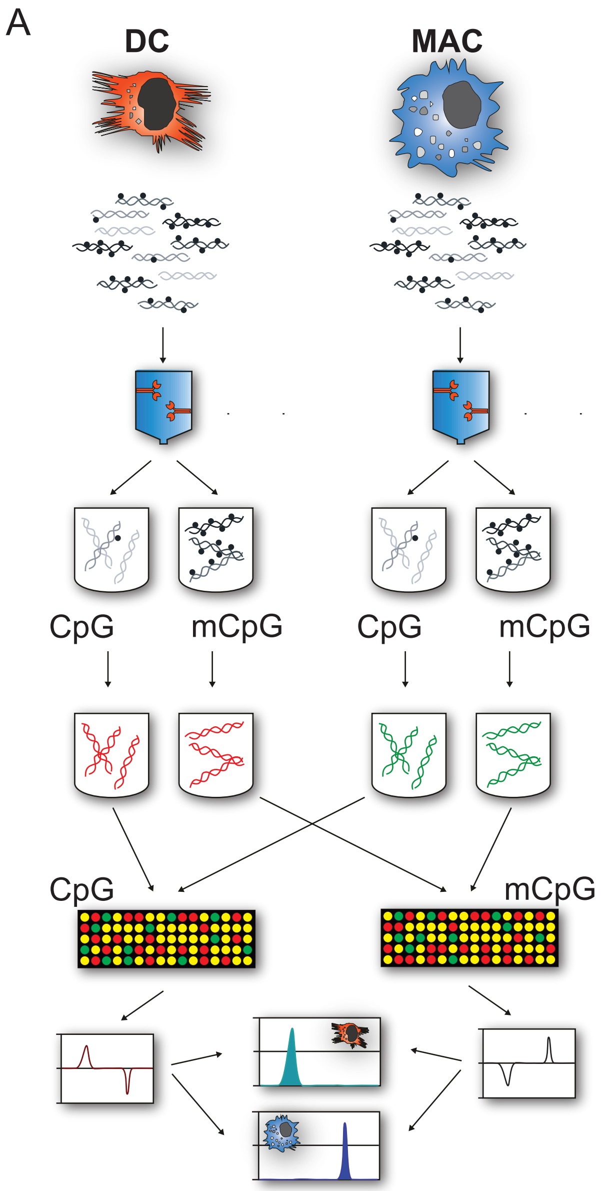
A
DC
MAC
CpG
mCpG
CpG
mCpG
CpG
mCpG

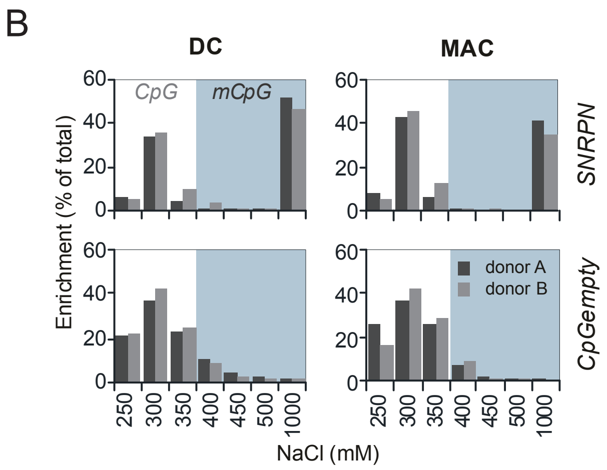
B
DC
MAC
CpG
mCpG
Enrichment (% of total)
SNRPN
CpGempty
donor A
donor B
0
20
40
60
250
300
350
400
450
500
1000
NaCl (mM)

Figure S3

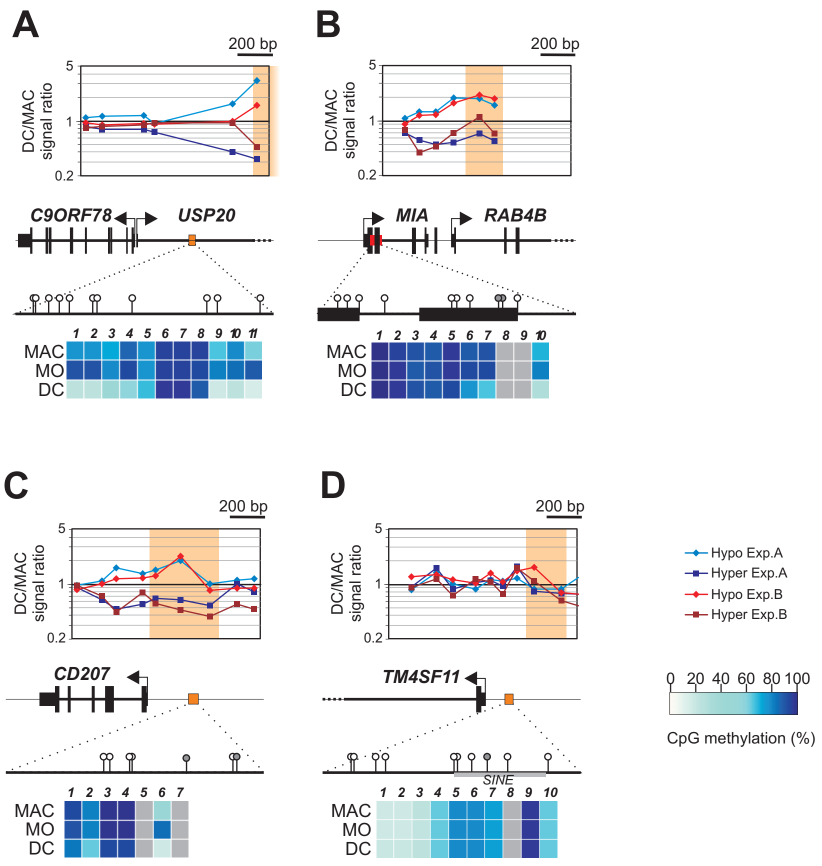
A
B
C
D
200 bp
DC/MAC signal ratio
5
1
0.2
C9ORF78
USP20
MIA
RAB4B
CD207
TM4SF11
SINE
MAC
MO
DC
Hypo Exp.A
Hyper Exp.A
Hypo Exp.B
Hyper Exp.B
0 20 40 60 80 100
CpG methylation (%)

Figure S4

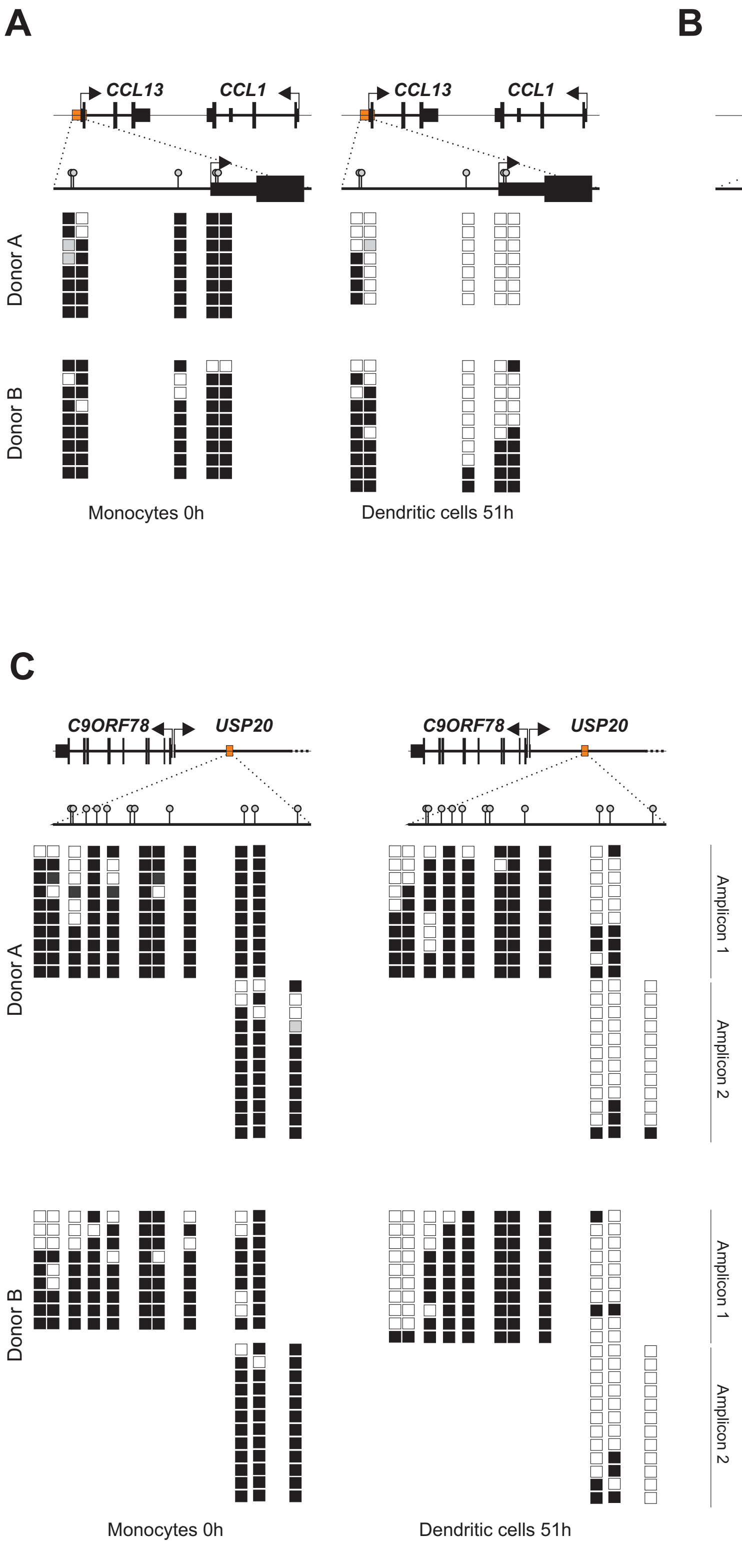

A
CCL13
CCL1
Donor A
Donor B
Monocytes 0h
Dendritic cells 51h
B
CD207
Donor A
Donor B
Monocytes 0h
Dendritic cells 51h
C
C9ORF78
USP20
Donor A
Donor B
Amplicon 1
Amplicon 2
Amplicon 1
Amplicon 2
Monocytes 0h
Dendritic cells 51h

Figure S5

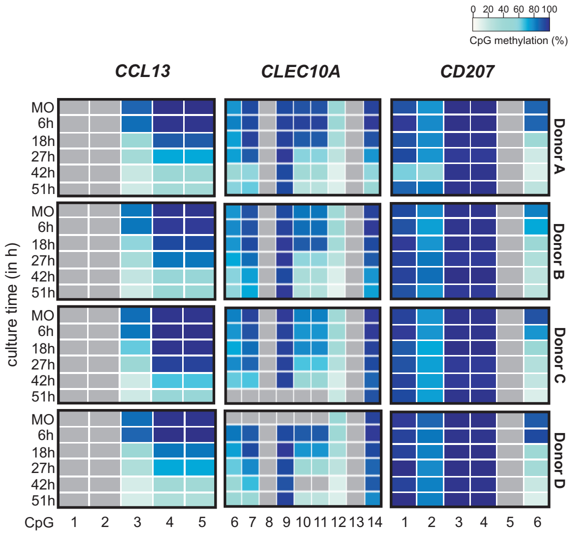

0 20 40 60 80 100
CpG methylation (%)
CCL13
CLEC10A
CD207
MO
6h
18h
27h
42h
51h
culture time (in h)
Donor A
Donor B
Donor C
Donor D
CpG 1 2 3 4 5
6 7 8 9 10 11 12 13 14
1 2 3 4 5 6

Figure S6

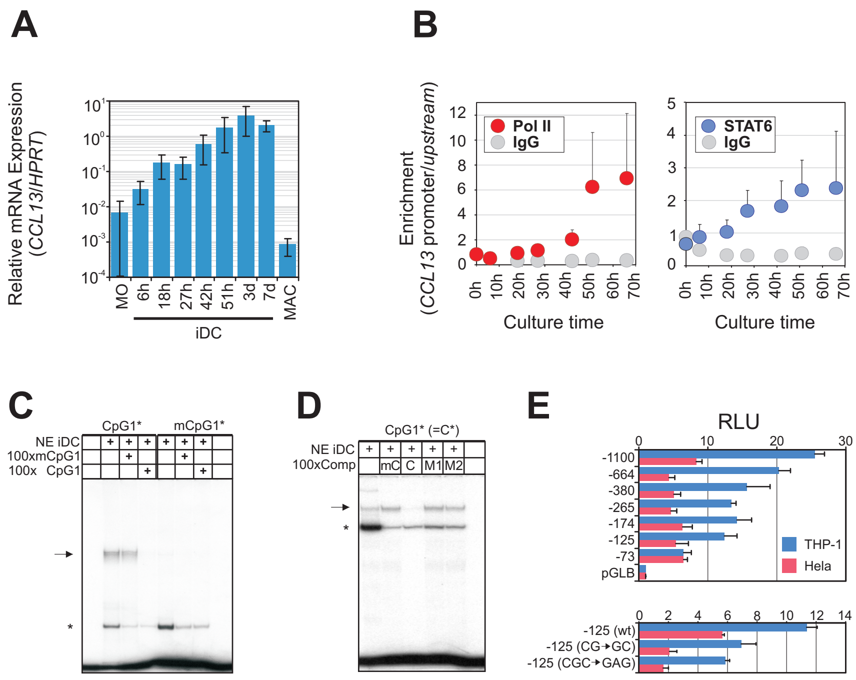

A
B
Relative mRNA Expression (CCL13/HPRT)
MO
6h
18h
27h
42h
51h
3d
7d
MAC
iDC
Enrichment (CCL13 promoter/upstream)
Pol II
IgG
STAT6
Culture time
C
CpG1*
mCpG1*
NE iDC
100xmCpG1
100x CpG1
D
CpG1* (=C*)
100xComp
mC
C
M1
M2
E
RLU
-1100
-664
-380
-265
-174
-125
-73
pGLB
THP-1
Hela
-125 (wt)
-125 (CG→GC)
-125 (CGC→GAG)

Figure S7

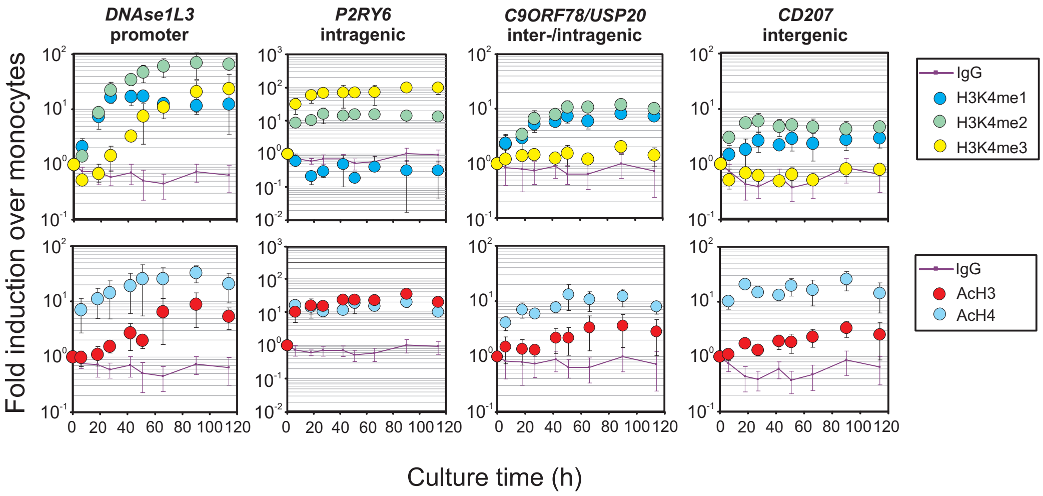
DNAse1L3
promoter
P2RY6
intragenic
C9ORF78/USP20
inter-/intragenic
CD207
intergenic
IgG
H3K4me1
H3K4me2
H3K4me3
IgG
AcH3
AcH4
Fold induction over monocytes
Culture time (h)

Figure S8

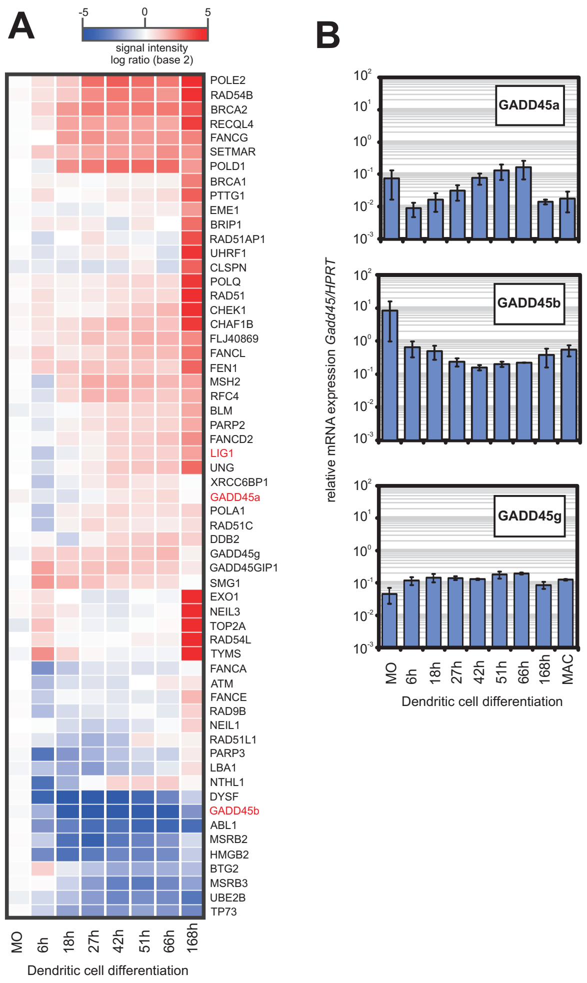
A
-5
0
5
signal intensity
log ratio (base 2)
POLE2
RAD54B
BRCA2
RECQL4
FANCG
SETMAR
POLD1
BRCA1
PTTG1
EME1
BRIP1
RAD51AP1
UHRF1
CLSPN
POLQ
RAD51
CHEK1
CHAF1B
FLJ40869
FANCL
FEN1
MSH2
RFC4
BLM
PARP2
FANCD2
LIG1
UNG
XRCC6BP1
GADD45a
POLA1
RAD51C
DDB2
GADD45g
GADD45GIP1
SMG1
EXO1
NEIL3
TOP2A
RAD54L
TYMS
FANCA
ATM
FANCE
RAD9B
NEIL1
RAD51L1
PARP3
LBA1
NTHL1
DYSF
GADD45b
ABL1
MSRB2
HMGB2
BTG2
MSRB3
UBE2B
TP73
MO
6h
18h
27h
42h
51h
66h
168h
Dendritic cell differentiation
B
relative mRNA expression Gadd45/HPRT
GADD45a
GADD45b
GADD45g
10^2
10^1
10^0
10^{-1}
10^{-2}
10^{-3}
MO
6h
18h
27h
42h
51h
66h
168h
MAC
Dendritic cell differentiation
