# Supplementary material for: Active DNA demethylation in human postmitotic cells correlates with activating histone modifications, but not transcription levels
Source: Genome Biol. 2010 Jun 18;11(6):R63. doi: 10.1186/gb-2010-11-6-r63 (PMC2911111; doi:10.1186/gb-2010-11-6-r63)
Supplement: Additional file 2 — Oligonucleotides for bisulfite amplicon generation. Genomic locations and oligonucleotides for EpiTYPER bisulfite amplicons. [file gb-2010-11-6-r63-S2.DOC]

**Additional Table S1**

**Oligonucleotides for bisulfite amplicon generation**

| **Amplicon** | **Chromosomal location (hg17)** | **Sense** | **Antisense** |
| --- | --- | --- | --- |
| Epi00100_SPI1.1 | chr11:47360676-47361039 | aggaagagagGATTGGGTTAGGGTTTTAGATAGGA | cagtaatacgactcactatagggagaaggctCCAAACCCCTTAAACTTAACCATAC |
| Epi00103_STAT5A.1 | chr17:37688826-37689055 | aggaagagagAGTTGTTTGGTTTTGTGTGTTTTTT | cagtaatacgactcactatagggagaaggctAAAAAAATCCTACTTCCTCTACCCA |
| Epi00104_STAT5A.2 | chr17:37689169-37689327 | aggaagagagAAAGTGATTTTTTTGAAGAGTGGTG | cagtaatacgactcactatagggagaaggctTCCAAAAAAACAAATCAAAACCTAA |
| Epi00105_SLC27A3.1 | chr1:150559763-150560058 | aggaagagagGAAGGGATTTGGTTTTGGTTATTAT | cagtaatacgactcactatagggagaaggctAAAAAAACATCTCTATCCCTCCCTA |
| Epi00106_SLC27A3.2 | chr1:150560101-150560536 | aggaagagagGGAGAGTTTTTTGGTTATGTTGTTG | cagtaatacgactcactatagggagaaggctAACCCCTAACCCATTTAATTCTACA |
| Epi00107_SLC27A3.3 | chr1:150560350-150560536 | aggaagagagGGAGAGTATTTGTTGGTTGGTTTTA | cagtaatacgactcactatagggagaaggctAACCCCTAACCCATTTAATTCTACA |
| Epi00108_STAT5A.1 | chr17:37689347-37689639 | aggaagagagTTTATAGGGAGGTATTAGGGTTTGG | cagtaatacgactcactatagggagaaggctTCCCTTCTTCAAAAAAAATTCCTAT |
| Epi00109_CCL13.1 | chr17:29707391-29707691 | aggaagagagTTTGTGGTTTGAATAGTTAGAAGGA | cagtaatacgactcactatagggagaaggctCAACAAACACAAAAACACTACAAAAA |
| Epi00110_CCL13.2 | chr17:29707330-29707746 | aggaagagagTTTATGGTTTTTTATGGTGAATGGT | cagtaatacgactcactatagggagaaggctAAAATAACTTACCTAACTAAACAAATCCC |
| Epi00111_P2RY6.1 | chr11:72661311-72661507 | aggaagagagTTTGGTTATGTTTGGAGTTTGTAGA | cagtaatacgactcactatagggagaaggctAAAAAAATACCCTTACCAACCATTT |
| Epi00112_P2RY6.2 | chr11:72661477-72661974 | aggaagagagGAGTGTAAATGGTTGGTAAGGGTAT | cagtaatacgactcactatagggagaaggctAAATCCCAAATATCTTCAAAAAACC |
| Epi00116_CD207.2 | chr2:70976696-70977195 | aggaagagagATTTTTGGATTTTTATGTTTGGGAT | cagtaatacgactcactatagggagaaggctAACCCAAAATTCCATACCTTTACTC |
| Epi00117_CBR3.1 | chr21:36426706-36427125 | aggaagagagAGTTGATTGGTGAGTATGGGTTTTA | cagtaatacgactcactatagggagaaggctAAAATTAACCACCCCAATAAAAAAA |
| Epi00123_DNASE1L3.1 | chr3:58171512-58171736 | aggaagagagTTTTTTAGGAAAGGGGTTTATTTTT | cagtaatacgactcactatagggagaaggctAAAATCCAACACTCCAAACACTACT |
| Epi00124_DNASE1L3.2 | chr3:58171637-58172079 | aggaagagagGGAGGAGAAGTAGTAGTGGGGTTAG | cagtaatacgactcactatagggagaaggctCACCCCAAATACCCTCTAAAATAAA |
| Epi00125_DNASE1L3.3 | chr3:58172052-58172297 | aggaagagagTGGTTTATTTTAGAGGGTATTTGGG | cagtaatacgactcactatagggagaaggctTCTCTAACAACACACTCCTAATATTTATAC |
| Epi00128_C14ORF166B.1 | chr14:76361834-76362145 | aggaagagagTTGATTTGATTATTGATGTTTTGAA | cagtaatacgactcactatagggagaaggctATCAATTTTCTTCTACCCAACTCTTC |
| Epi00129_C14ORF166B.2 | chr14:76362120-76362472 | aggaagagagGAAGAGTTGGGTAGAAGAAAATTGAT | cagtaatacgactcactatagggagaaggctAACTCAAAAACCACTTTCATTTCAT |
| Epi00131_CHI3L1.1 | chr1:199887419-199887883 | aggaagagagGTAGAGTAGGGTAGGGTGTGGTTTT | cagtaatacgactcactatagggagaaggctTTCCACCTAACCAAAAACCTAAAAT |
| Epi00132_CHI3L1.2 | chr1:199888000-199888353 | aggaagagagGTTTTTAGGTTGGGTAAGGGTTAGA | cagtaatacgactcactatagggagaaggctCATCAAACTTAAATTCCAAAACCTC |
| Epi00133_CHI3L1.3 | chr1:199888136-199888582 | aggaagagagAGAGGGAAAGATAGGGAAATTTTTA | cagtaatacgactcactatagggagaaggctTCTTAAAAAACCCTTAAACCCATTC |
| Epi00136_CCL13.2.1 | chr17:29698090-29698506 | aggaagagagTTGTGATTTTGTGTTAATATTGAGTGT | cagtaatacgactcactatagggagaaggctTCTTACAAACCAAACAAAAATAAACC |
| Epi00143_MIA.2 | chr19:45973357-45973709 | aggaagagagGGTGGTTTTATGTTTAAGTTGGTTG | cagtaatacgactcactatagggagaaggctCCTCAAATCTTCCCTTCATAAAAAT |
| Epi00147_C9ORF78.2 | chr9:129680831-129681232 | aggaagagagAGAGGTTTTTGTGAGGAAGTTTTTT | cagtaatacgactcactatagggagaaggctACTACCCACACACTTCTATATCTCCTC |
| Epi00148_C9ORF78.3 | chr9:129681085-129681304 | aggaagagagGGAATTTTGTTATTTTTTAGGGTGG | cagtaatacgactcactatagggagaaggctAAAACCACCATCCTCTAACTCTC |
| Epi00150_ZNF642.1 | chr1:40612628-40612847 | aggaagagagTTTTGGTTTTATTTGGGTTTAAGGT | cagtaatacgactcactatagggagaaggctAAAACTTATAAAAAATATCCCACCCC |

**Additional Table S1 (continued)**

**Oligonucleotides for bisulfite amplicon generation**

| **Amplicon** | **Chromosomal location (hg17)** | **Sense** | **Antisense** |
| --- | --- | --- | --- |
| Epi00153_ADPGK.1 | chr15:70865888-70866369 | aggaagagagAGGTTATGTTTAAAGGTTAGAGTTAGAGT | cagtaatacgactcactatagggagaaggctCTCATAAATCCCTACCAAACAAAAA |
| Epi00154_ADPGK.2 | chr15:70866344-70866636 | aggaagagagGTTTTTGTTTGGTAGGGATTTATGA | cagtaatacgactcactatagggagaaggctATTAAAAAACAAACTTCCCATTTCC |
| Epi00156_NDRG2.1 | chr14:20560152-20560582 | aggaagagagATTGAAATAGTGGTTGGAAGTAAGA | cagtaatacgactcactatagggagaaggctCACTCCCAACTCCCTAACCTTAATA |
| Epi00159_RAP1GAP.2 | chr1:21742512-21742970 | aggaagagagTAGTTTTATAGGGGTTGGGGATTAG | cagtaatacgactcactatagggagaaggctTCCTAAACAAAAAATCAAAAAACCC |
| Epi00162_MMP7.1 | chr11:101906504-101906707 | aggaagagagGGAATTTTAAGTAAGTGGGTTGTGA | cagtaatacgactcactatagggagaaggctACAATCACTAACAAAAAACACCAAA |
| Epi00165_CCL17.1 | chr16:55995686-55995962 | aggaagagagTTGAGAATATATTGTAGGGGGTAAGG | cagtaatacgactcactatagggagaaggctCCCCCAAATCTAAAACTAAATTTCT |
| Epi00166_CCL17.2 | chr16:55995938-55996246 | aggaagagagAGAAATTTAGTTTTAGATTTGGGGG | cagtaatacgactcactatagggagaaggctATTTTAAATTCAACTCTCCCATCAA |
| Epi00167_CCL17.3 | chr16:55996175-55996657 | aggaagagagTTGAGGTTTAGAGAGAAGTGATTTTG | cagtaatacgactcactatagggagaaggctAACACCTCCCTCATCAACTACATAC |
| Epi00170_BACH2.1 | chr6:91064223-91064594 | aggaagagagGTGTTAGTGTTGTGTTGGTGTTTGT | cagtaatacgactcactatagggagaaggctCAACACCTTCAACTTACTTTCAACC |
| Epi00171_BACH2.2 | chr6:91064569-91064757 | aggaagagagTGGTTGAAAGTAAGTTGAAGGTGTT | cagtaatacgactcactatagggagaaggctTTTATCCTAAAAAACACCAAACCAA |
| Epi00172_BACH2.3 | chr6:91064808-91065207 | aggaagagagAATTTTTGGTTTGTTTTGGTTAGGT | cagtaatacgactcactatagggagaaggctTCTCAAAATAAAAAAACTCCAATCT |
| Epi00176_PLLP.1 | chr16:55876614-55876899 | aggaagagagGAAAGTAAAGAAGAATTTTGGGAGATT | cagtaatacgactcactatagggagaaggctAACCTCCCATCTTCTAAATAACCCC |
| Epi00178_KIAA0430.1 | chr16:15643925-15644108 | aggaagagagTTAGTGAGAGTGGTTGAAGTTTTAGA | cagtaatacgactcactatagggagaaggctCCCTAATAAAACCCTCCAAAAATAA |
| Epi00179_KIAA0430.2 | chr16:15644075-15644307 | aggaagagagTTGTGTATTTTATTTTTGGAGGGTT | cagtaatacgactcactatagggagaaggctAAAAACAATATCCCCTCTTTCCC |
| Epi00181_TPP2.1 | chr13:102047685-102047886 | aggaagagagGGGGTGGGTAGAGGTTAGAGTTAG | cagtaatacgactcactatagggagaaggctAAAAAATCCCTAAAACCAAAAAAAA |
| Epi00182_TPP2.2 | chr13:102047848-102048054 | aggaagagagGGGGAAGTTTGGGTTTTTTTT | cagtaatacgactcactatagggagaaggctTTACCTATCTAATATCTCCACCCCA |
| Epi00184_CLEC10A.1 | chr17:6923689-6924152 | aggaagagagGAAGATAAGGTTGGAAATGGGTTAT | cagtaatacgactcactatagggagaaggctACCTCTAATCCTTACAACACAACCA |
| Epi00185_CLEC10A.2 | chr17:6924086-6924365 | aggaagagagTATTATTATTTGTGGGAGGTTTGGA | cagtaatacgactcactatagggagaaggctAACAAAACTAACCTCAAACCCAACT |
| Epi00188_MAPKAPK3.1 | chr3:50624882-50625081 | aggaagagagGGGTGTAGAGGATAGTTTTAGAAATGA | cagtaatacgactcactatagggagaaggctCTCTTCTCCCCTAACTAACAAAACC |
| Epi00189_MAPKAPK3.2 | chr3:50625057-50625555 | aggaagagagGGTTTTGTTAGTTAGGGGAGAAGAG | cagtaatacgactcactatagggagaaggctATAAACCCTACAACCTCTCCAAATC |
| Epi00191_TRIM15.1 | chr6:30238807-30239292 | aggaagagagGGTTATTATGGGTAGATGTGGTGAG | cagtaatacgactcactatagggagaaggctAATACCAACACAAAAAAACCACCTA |
| Epi00192_TRIM15.2 | chr6:30239051-30239544 | aggaagagagAAGGTGTGTTTATAGGGAATGGTTA | cagtaatacgactcactatagggagaaggctCCAACCACTATAATCTACAAATTCCA |
