# Supplementary material for: Active DNA demethylation in human postmitotic cells correlates with activating histone modifications, but not transcription levels
Source: Genome Biol. 2010 Jun 18;11(6):R63. doi: 10.1186/gb-2010-11-6-r63 (PMC2911111; doi:10.1186/gb-2010-11-6-r63)
Supplement: Additional file 4 — Oligonucleotide sequences used for quantitative PCR, cloning, and electrophoretic mobility shift assay. [file gb-2010-11-6-r63-S4.DOC]

**Supplementary Table S3**

**Oligonucleotide sequences**

| **Gene** | **Oligonucleotide sequence** | **Application** |
| --- | --- | --- |
| **CCL13** | Forward: 5’-GAAGATCTCCTTGCAGAGGCT-3’  Reverse: 5’-GGGTCAGCACAGATCTCCTTG-3' | cDNA |
| **GADD45A** | Forward: 5’-TCCTGCTCTTGGAGACCGAC-3’  Reverse: 5’-GACTTAAGGCAGGATCCTTCCATTGAG-3’ | cDNA |
| **GADD45B** | Forward: 5’-AGTCGGCCAAGTTGATGAATGTG-3’  Reverse: 5’-GATGAGCGTGAAGTGGATTTGCAG-3’ | cDNA |
| **GADD45G** | Forward: 5’-CAGGACACAGTTCCGGAAAGCA-3’  Reverse: 5’-ACACAGAAGGTCACATTGTCGG-3’ | cDNA |
| **HPRT** | Forward: 5’-AAGTTTGTTGTAGGATATGCCC-3’  Reverse: 5’-GAACATTGATAATTTTACTGGCG-3’ | cDNA |
|  |  |  |
| **CCL13** | Forward: 5’-CTAGAAAAGTCTTTGGTGCCCAG-3’  Reverse: 5’-CTTGGAACTCTCAGAGGACCTTG-3’ | ChIP |
| **CCL13 up** | Forward: 5’-CAGACAAGCCTTATCGGTATCACCT-3’  Reverse: 5’-AATCGCTATCTCATTACGATGTTGGG-3’ | ChIP |
| **CD207** | Forward: 5’-TGCCCTCTCATTGGTCCCAG-3’  Reverse: 5’-TACCTACCTCAGCCTGCATTTCCA-3’ | ChIP |
| **CLEC10A** | Forward: 5’-TCTCCCTGCTTCCTCTGACATCC-3’  Reverse: 5’-AATCACACCCTCCAGACCTCCC-3’ | ChIP |
| **DNASE1L3** | Forward: 5’-GGGCTCACCTTCACAATGACATCC-3’  Reverse: 5’-TCTGCTCCTTCAACGTCAGGTC-3’ | ChIP |
| **CpGempty** | Foward: 5'-GAAACCCTCACCCAGGAGATACAC-3'  Reverse: 5'-TGCAGTGGGACTTTATTCCATAGAAGAG-3' | MCIp |
| **P2RY6** | Forward: 5’-CTGCTGACACCGACACAGAG-3’  Reverse: 5’-GAGCTGGCAAGAGATGAGAGTCC-3’ | ChIP |
| **SNRPN** | Forward: 5'-TACATCAGGGTGATTGCAGTTCC-3'  Reverse: 5'-TACCGATCACTTCACGTACCTTCG-3' | MCIp |
| **STAT5** | Forward: 5’-GCACAGACTCTGCATCCTCTTCTC-3’  Reverse: 5’-AGTCCTGCTTCCTCTGCCCA-3’ | ChIP |
| **USP20** | Forward: 5'-AGTACGCGTGTTTGGATTTGGG-3'  Reverse: 5'-TGCTCTGAACACTCAGCTCCAC-3' | ChIP |

**Supplementary Table S3 (continued)**

**Oligonucleotide sequences**

| **Gene** | **Oligonucleotide sequence** | **Application** |
| --- | --- | --- |
| **CpG1 (-80 bp)** | Forward: 5’-CATCATGACTTGGTCAACGCCCTGCTCA-3’ | EMSA |
| **CpG1 M1** | Forward: 5’-CATCATGACTTGGTCAA**GC**CCCTGCTCA-3’ | EMSA, Cloning |
| **CpG1 M2** | Forward: 5’-CATCATGACTTGGTCAA**GAG**CCTGCTCA-3’ | EMSA, Cloning |
|  |  |  |
| **CCL13P_-73** | Forward: 5'-GAACAGCTAGCTCAGGCCAAGGTCCTCTG-3' | Cloning |
| **CCL13P_-125** | Forward: 5'-GAACAGCTAGCCAGCTCAGCAGATTCAGG-3' | Cloning |
| **CCL13P_-174** | Forward: 5'-GAACTGCTAGCCCTCAGCTTCCCTCTTGC-3' | Cloning |
| **CCL13P_-265** | Forward: 5'-GAACTGCTAGCGTGAATGGCTGGGGCG-3' | Cloning |
| **CCL13P_-380** | Forward: 5'-ACGTGAACAGAGTCCTTAGCACAG-3' | Cloning |
| **CCL13P_-664** | Forward: 5'-GAACAGCTAGCACAATATGTAATGTAAGGAGCCC-3' | Cloning |
| **CCL13P_-1100** | Forward: 5'-TCAACGCTAGCTTAATTATAGGCTACAGAACCAG-3'  Reverse: 5'-AGTCTCCAGATCTTTGCCTCTCTGCTCCTC-3' | Cloning |
|  |  |  |
| **ENH_STAT5** | Forward: 5’-tctacCTGCAGACTCTGCATCCTCTTCTCTTCGT-3’  Reverse: 5’-tctacACTAGTTTCCTGTGCTCCAAGTCCCT-3’ | Cloning |
| **ENH_CD207** | Forward: 5’-tctacCTGCAGCTCCACATCACTGCCTGCCT-3’  Reverse: 5’-tctacACTAGTCTTCTGACTCCCTCCCTCTCCA-3’ | Cloning |
| **ENH_CBR3** | Forward: 5’-tctacCTGCAGCATAGACGGGCAAGTGGAG-3’  Reverse: 5’-tctacACTAGTCCTGGCCTAGTTCTTCTTGAGTGAC-3’ | Cloning |
| **ENH_ADPGK** | Forward: 5’-tctacCTGCAGCAAAGGCCAGAGTCAGAGCAC-3’  Reverse: 5’-tctacACTAGTGCAAACTTCCCATTTCCTTCCAAACC-3’ | Cloning |
| **ENH_RAP1GAP** | Forward: 5’-tctacCTGCAGCCCAAGGTCCACAGCAAGTGAG-3’  Reverse: 5’-tctacACTAGTTCTACAGACCGCTCCTGCAC-3’ | Cloning |
